# Fabrication and Characterization of Roll-to-Roll Printed Air-Gap Touch Sensors

**DOI:** 10.3390/polym11020245

**Published:** 2019-02-02

**Authors:** Sang Hoon Lee, Sangyoon Lee

**Affiliations:** 1 Department of Mechanical Design and Production Engineering, Konkuk University, Seoul 05029, Korea; hoon1911@konkuk.ac.kr; 2 School of Mechanical Engineering, Konkuk University, Seoul 05029, Korea

**Keywords:** air-gap, roll-to-roll printed electronics, capacitive touch sensor, sacrificial layer

## Abstract

Although printed electronics technology has been recently employed in the production of various devices, its use for the fabrication of electronic devices with air-gap structures remains challenging. This paper presents a productive roll-to-roll printed electronics method for the fabrication of capacitive touch sensors with air-gap structures. Each layer of the sensor was fabricated by printing or coating. The bottom electrode, and the dielectric and sacrificial layers were roll-to-roll slot-die coated on a flexible substrate. The top electrode was formed by roll-to-roll gravure printing, while the structural layer was formed by spin-coating. In particular, the sacrificial layer was coated with polyvinyl alcohol (PVA) and removed in water to form an air-gap. The successful formation of the air-gap was verified by field emission scanning electron microscopy (FE-SEM). Electrical characteristics of the air-gap touch sensor samples were analyzed in terms of sensitivity, hysteresis, and repeatability. Experimental results showed that the proposed method can be suitable for the fabrication of air-gap sensors by using the roll-to-roll printed electronics technology.

## 1. Introduction

Printed electronics is a technology for fabricating electronic devices using printing methods, such as inkjet, screen, and gravure printings. In addition to printing, coating processes such as spin-coating and slot-die coating can be also used [1,2,3]. Printed electronics are considered more advantageous in terms of productivity and flexibility than the conventional semiconductor technology [4,5]. Examples of printed electronic devices include organic thin film transistors [6,7,8,9], organic light-emitting diodes [10,11], and sensors [12,13,14].

Sensors with air-gap structures are used in numerous applications, such as capacitive touch sensors [15], pressure sensors [16], accelerometers [17], and gyroscopes [18,19]. Their fabrication is usually based on the semiconductor process where the etching process is used to fabricate devices with air-gap structures such as cantilever, bridge, and comb-drive [20,21,22]. On the other hand, the printed electronics method does not employ the etching process, and thus it is difficult to fabricate devices with air-gap structures. Therefore, a novel printed electronics method suitable for the mass-production of air-gap-based electronic devices is needed.

A few studies have been carried out to fabricate air-gap-based devices using printed electronics methods. Some of them reported methods for the formation of air-gaps by printing a sacrificial layer and chemical decomposition of the layer [23,24]. Subramanian et al. fabricated a cantilever structural reed relay device [23]. An air-gap structure was formed by inkjet printing and chemical decomposition of poly(methyl methacrylate) (PMMA) sacrificial layer. Clement et al. fabricated a cantilever structural gas sensor using screen printing [24]. An air-gap structure was formed by screen printing of epoxy-strontium carbonate (SrCO_3_) sacrificial layer and the layer was chemically decomposed.

In other studies, air-gaps were formed by bonding the upper and lower parts of electronic devices [25,26]. Kim et al. fabricated a bridge structural pressure sensor by inkjet printing [25]. An air-gap structure was formed by assembling an upper film on a lower film. Kanazawa et al. also fabricated a cantilever structural strain sensor with similar way [26]. An air-gap structure was formed by transferring a cantilever beam (upper part) to a substrate (lower part) using a polydimethylsiloxane (PDMS) dummy layer. The proposed methods are focused only on the formation of air-gaps and are not suitable for productive fabrication of air-gap devices.

**Figure 1:**
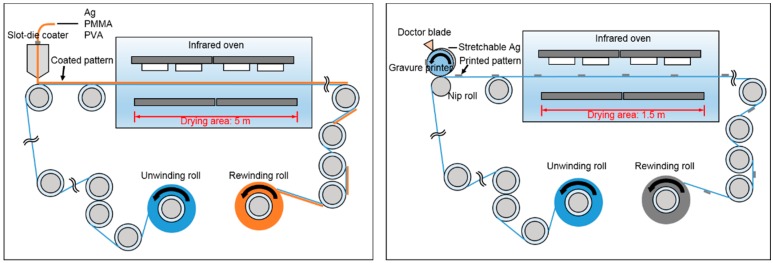
Schematics of the roll-to-roll slot-die coater (**left**) and gravure printer (**right**).

For a productive fabrication, the roll-to-roll process is known as the most promising among the printed electronics processes [27,28,29]. Figure 1 shows the schematics of the roll-to-roll processes. After unwinding of a flexible substrate, patterns are printed or coated on the substrate, and then dried. The patterned substrate is then rewound. The roll-to-roll process is a continuous one and does not stop during printing and drying. If printing/coating speed and viscosity of materials are not set properly, materials cannot be transferred to the substrate successfully, and so printability or coatability deteriorates [30]. If inappropriate drying condition of process and materials is applied, printed patterns will not dry before rewinding, and they will spread eventually [31]. For a high printability or coatability of patterns, the roll-to-roll process conditions and materials should be set carefully.

**Figure 2:**
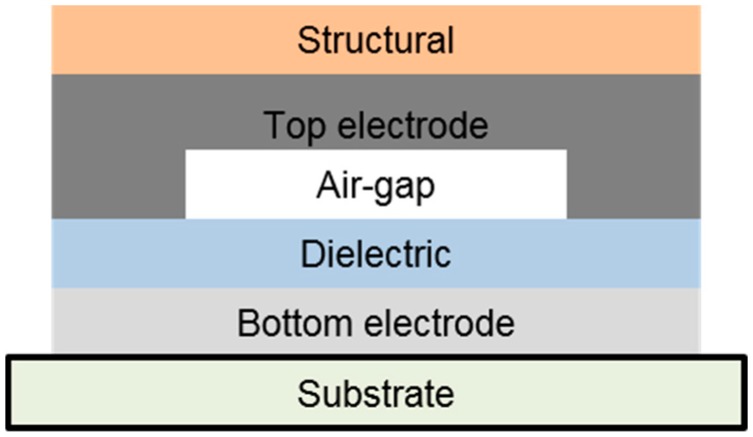
Schematic of the air-gap touch sensor.

In this study, we propose a roll-to-roll printed electronics method for the fabrication of air-gap sensors. Roll-to-roll printing and coating processes were used for the fabrication of capacitive air-gap touch sensors on a flexible substrate. Figure 2 shows the layered structure of the sensor. The capacitive air-gap touch sensor was designed to sense touch following the characteristics of capacitors. When an external force is applied to the touch sensor, the gap between the bottom electrode and the top electrode decreases, and hence capacitance increases. When the force is released, the top electrode returns to the original form and capacitance also returns to the initial value. The materials and methods used in the fabrication are described in detail in Section 2. The fabrication and test results along with a discussion are presented in Section 3, which is followed by concluding remarks in Section 4.

## 2. Materials and Methods 

Capacitive air-gap touch sensor samples were fabricated on a flexible poly(ethylene terephthalate) (PET) substrate (SH34, SKC) with printing and coating processes. The sensor samples are composed of five layers, as shown in Figure 2. The materials and fabrication processes for each layer of the sensor are summarized in Table 1. The process conditions and materials of the roll-to-toll printed electronics method were determined through repeated experiments.

### 2.1. Bottom Electrode

**Figure 3:**
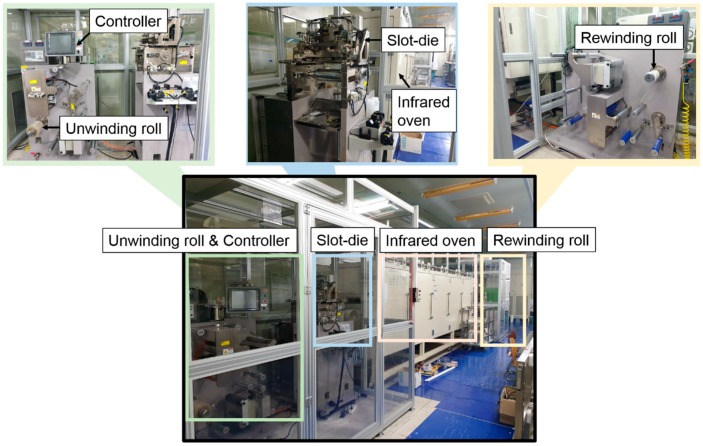
Roll-to-roll slot-die coater.

A silver ink (TEC-CO-021, InkTec, Ansan, Korea) was coated on the PET substrate with a roll-to-roll slot-die coater (Figure 3) at a speed of 0.5 m/min. The bottom electrode was then dried for 10 min at 100 °C in an infrared oven installed in the slot-die coater.

### 2.2. Dielectric Layer

As shown in Figure 2, an air-gap is designed between the bottom and top electrodes. If an external force is applied to the sensor, the air-gap decreases, and the two electrodes can short. In order to prevent the problem, a dielectric layer was coated on the bottom electrode.

Poly(methyl methacrylate) (PMMA; Sigma Aldrich, MO, USA) with a molecular weight (MW) of 120,000 was used for the dielectric layer. A PMMA powder (10 wt %) was dissolved in acetone. The solution of PMMA was roll-to-roll slot-die coated on the bottom electrode to form the dielectric layer. The coating speed was 0.5 m/min. The dielectric layer was then dried for 10 min at 70 °C in the infrared oven.

### 2.3. Sacrificial Layer

**Figure 4:**
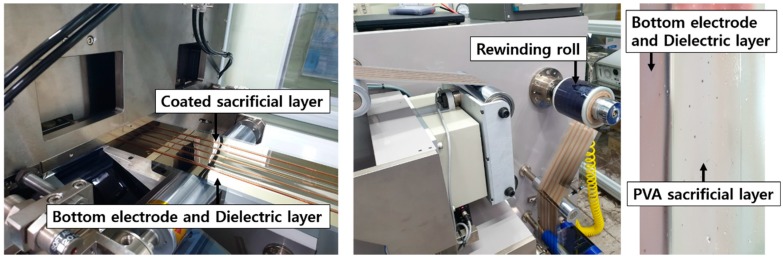
Roll-to-roll slot-die coating process: a coated sacrificial layer (**left**); rewinding of the sacrificial layer (**middle**); and of a sample of the sacrificial layer (**right**).

Polyvinyl alcohol (PVA; Comscience; Gwangju, Korea) was chosen as a material for the sacrificial layer as it is decomposable and removable in water [32,33]. A PVA powder (15 wt %) was dissolved in water. The solution of PVA was roll-to-roll slot-die coated at a speed of 0.5 m/min. Figure 4 shows the coating of the sacrificial layer. The sacrificial layer with a width of 10 mm was dried for 10 min at 70 °C in the infrared oven.

### 2.4. Top Electrode

The top electrode should easily deform when an external force is applied to the sensor and should rapidly restore to its original state when the force is released. Considering this requirement, a stretchable silver ink (SSP 2801, Toyobo, Osaka, Japan) was chosen as a suitable material for the top electrode. The stretchable silver ink has advantages over the general silver ink in terms of high restoration and elastic force and smaller probabilities of cracking and breakage [34,35].

**Figure 5:**
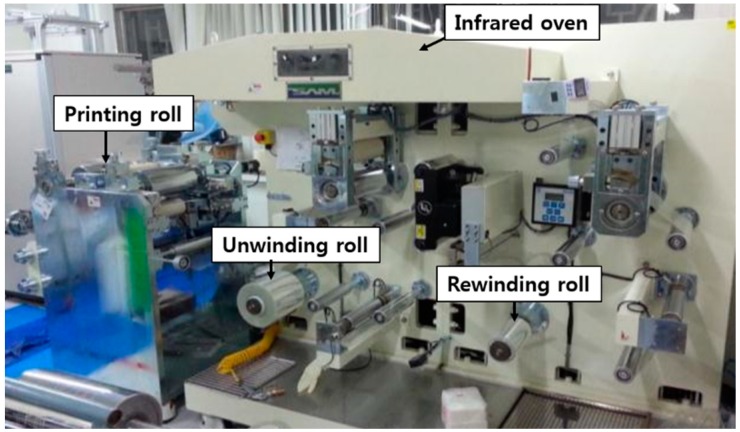
Roll-to-roll gravure printer.

**Figure 6:**
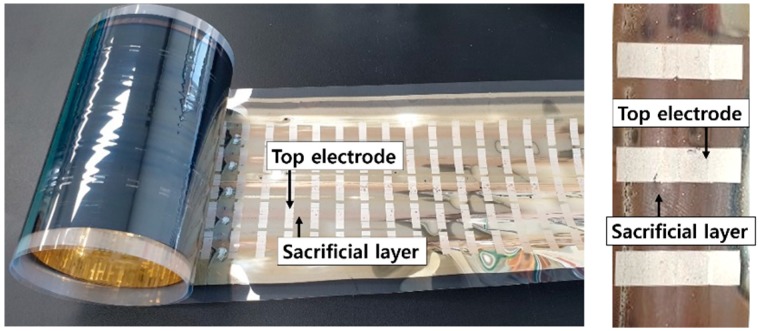
Roll-to-roll gravure printed top electrode on a PET roll (**left**); and enlarged image (**right**).

Unlike the processes for the previous layers, the top electrode was formed by printing such that a particular pattern could be fabricated. Stretchable silver ink was printed using a roll-to-roll gravure printer (Figure 5) at a speed of 3 m/min. The top electrode was dried for 30 s at 150 °C in the infrared oven, which is a part of the roll-to-roll gravure printer. A sample of the top electrode after the printing and curing is shown in Figure 6.

### 2.5. Structural Layer

In order to enhance the performance of the sensor, another layer referred to as structural layer was added. The structural layer helps the touch sensor to endure repeated force and restore the top electrode more quickly after the force is released. Epoxy resin (F-301, Alteco, Osaka, Japan) was spin-coated for 1 min at a speed of 3000 rpm to form the structural layer. It was then dried for 10 min at 150 °C on a hot-air oven.

### 2.6. Removal of the Sacrificial Layer

Sensor samples were put in a distilled water bath for 6 h in order to remove the PVA sacrificial layer and form an air-gap. The samples were taken out of the water bath and dried for 12 h at room temperature. Due to the restoration force of the top electrode and the structural layer, the top electrode does not collapse even after removal of the sacrificial layer. As a result, thickness of the air-gap is kept similar to that of the sacrificial layer.

## 3. Results and Discussion

Field emission scanning electron microscope (FE-SEM; SU8010, Hitachi, Tokyo, Japan) images were acquired to reveal the shape and thickness of the air-gap of the sensor samples and identify possible contact between the top electrode and dielectric layer. For measurement of the electrical characteristics of the sensor samples, three sets of experiments were carried out using equipment composed of a push-pull gauge, LCR meter, characterization system, and probe station.

### 3.1. Air-Gap Structure and Thickness of the Touch Sensor Samples

**Figure 7:**
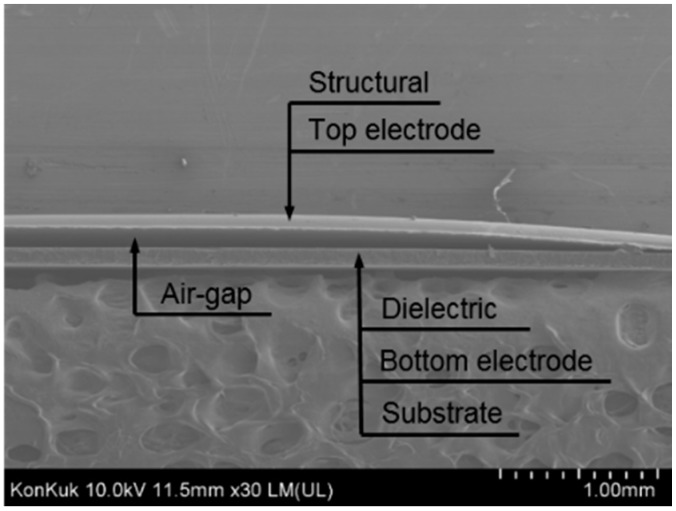
FE-SEM image of an air-gap touch sensor sample.

FE-SEM images of five sensor samples were acquired; an example is shown in Figure 7. The thickness of the air-gap was measured using a built-in function of dimension measurement in the FE-SEM system. The touch sensor was designed to have a bridge structure with an air-gap width of 10 mm; neither residues of PVA nor contact between the top electrode and dielectric layer were observed in the samples.

The thickness of the air-gap was compared to that of the sacrificial layer. The thickness of the air-gap should not significantly change during the removal process of the sacrificial layer. A large difference in thickness between the sacrificial layer and the air-gap can cause a large deviation in electrical performance and low yield.

**Figure 8:**
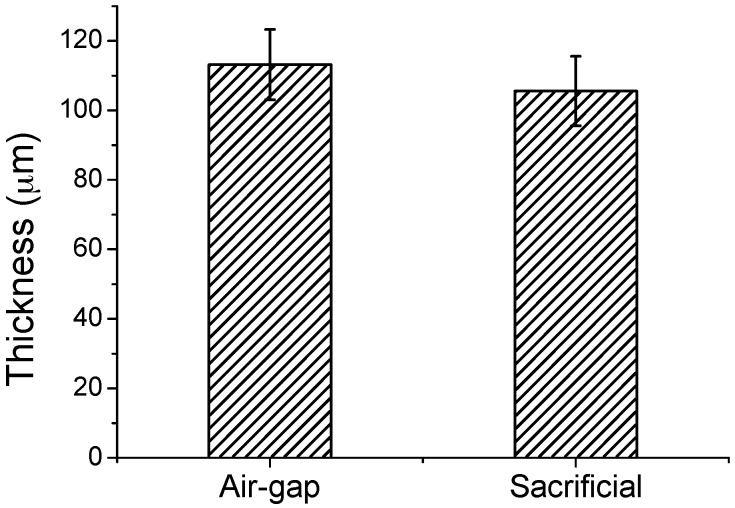
Comparison of the thicknesses of the air-gap and the sacrificial layer.

The thicknesses of the sacrificial layers of the five samples were measured from the FE-SEM images. As shown in Figure 8, the average thickness of the air-gap was 113.2 μm, while that of the sacrificial layer was 105.6 μm, which corresponds to an increase of 7.2%. The thickness of the air-gap was slightly increased owing to the expansion of the top electrode and structural layer by the buoyancy of water during the removal process of the sacrificial layer.

### 3.2. Electrical Characteristics of the Air-gap Touch Sensor Samples

**Figure 9:**
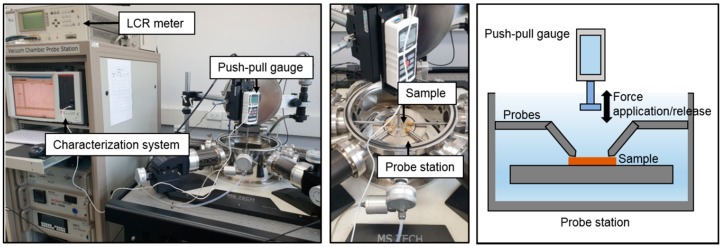
Measurement process of the electrical performance of the air-gap touch sensor: photograph of the entire equipment for measurement (**left**); enlarged photograph of the probe station and a sample (**middle**); and schematic of the measurement equipment (**right**).

Electrical characteristics of the air-gap touch sensor samples were investigated using a push-pull gauge (Series 5, Mark-10, NY, USA), LCR meter (4284A, Hewlett-Packard, CA, USA), characterization system (4200-SCS, Keithley, OH, USA), and probe station (M7VC, MS tech, Hwaseong, Korea), as shown in Figure 9.

First, the sensor sample was placed on the probe station and the probes were set to contact the bottom and top electrodes. The push-pull gauge was then automatically activated by setting the force magnitude, times of force application and release in the characterization system. The capacitance of the sensor was measured using the LCR meter and the characterization system. The measurement values of capacitance and force were saved in the characterization system.

The capacitance of a capacitor composed of two parallel plates can be theoretically calculated by:

(1) $$C=\varepsilon_{0}\cdot \varepsilon_{r}\cdot \frac{A}{d}$$

where *C* is the capacitance, $\varepsilon_{0}$ is the electric permittivity of vacuum, $\varepsilon_{r}$ is the relative permittivity of the dielectric, *A* is the area of the capacitor, and *d* is the thickness of the dielectric. As the dielectric of the sensor sample is the air-gap, $\varepsilon_{r}$ is close to 1. When an external force is applied to the touch sensor sample, the thickness of the air-gap decreases, and thus the capacitance increases. When the force is released, the top electrode returns to the original form and thus the capacitance returns to the initial value.

In this study, three sets of experiments were carried out to analyze the electrical characteristics of the touch sensor. Three samples were prepared for each set of experiments. The electrical characteristics were represented as a rate of capacitance change (Δ*C*/*C* (%)).

The touch sensor samples were compared to devices with polydimethylsiloxane (PDMS) as the dielectric layer. PDMS was often used as a material for the dielectric layer in other capacitance-type sensors fabricated by printed electronics methods [36,37,38,39]. PDMS has good elasticity and restoring force.

**Figure 10:**
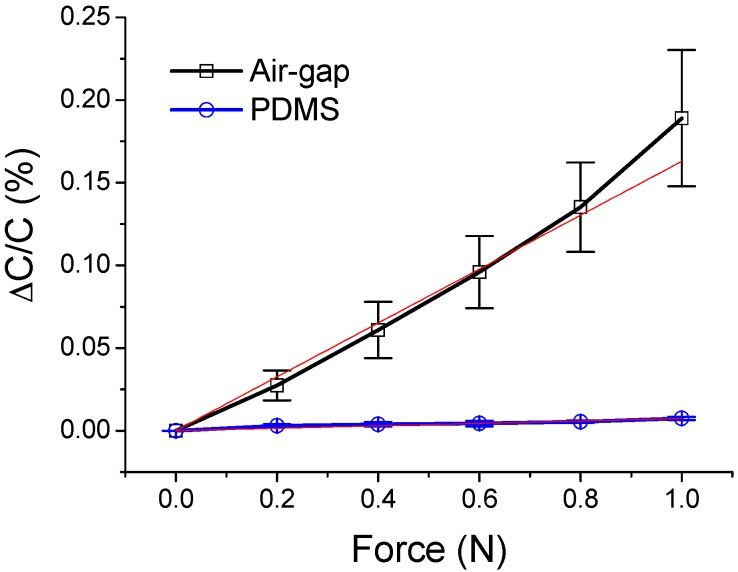
Relationships between the force and capacitance of the air-gap and PDMS touch sensors.

For the first set of experiments, three air-gap touch sensor samples and three PDMS-based touch sensor samples with thicknesses of 100 μm were prepared. For an accurate comparison, the PDMS-based sensor samples were fabricated using the same method as that for air-gap touch sensor sample. The capacitance was measured with the increase in the applied force in the range of 0 to 1 N with increments of 0.2 N. The measurement results are plotted in Figure 10.

When no force was applied, the average capacitance of the air-gap touch sensor samples was 366.47 pF. The average Δ*C*/*C* was 0.19% when the force of 1 N was applied. For the PDMS-based touch sensor samples, the average Δ*C*/*C* was 0.0075% when the same force was applied. Based on the least-squares linear regression method, the sensitivity of the air-gap touch sensor was 0.1630%/N, while that of the PDMS-based touch sensor was 0.0074%/N. These results imply that the air-gap touch sensor samples have significantly higher sensitivities than those of the PDMS-based sensor samples.

**Figure 11:**
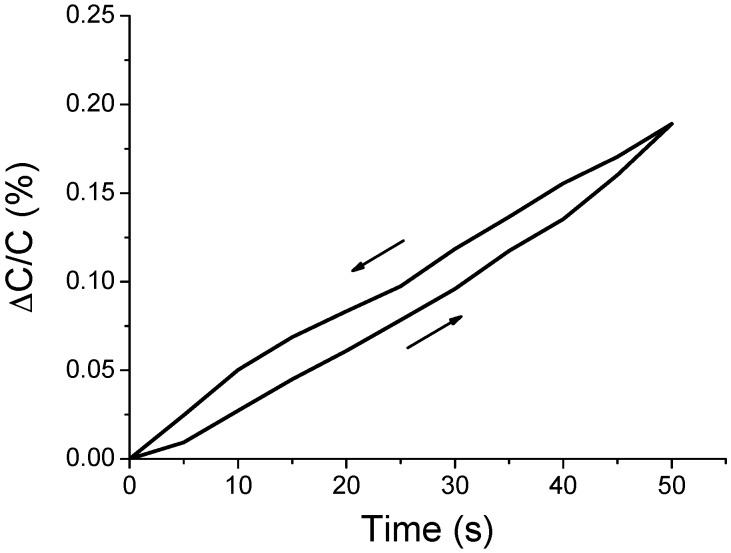
Capacitance change of the air-gap touch sensor upon the application and release of the force of 1 N.

The second set of experiments was carried out to reveal the hysteresis characteristics of the air-gap sensor samples. The capacitances of the sensor samples were measured with the increase in the force in the range of 0 to 1 N at a rate of 0.02 N/s, followed by decrease to 0 at the same rate. The results are shown in Figure 11.

The hysteresis error is calculated by Equations (2) and (3) [40,41]:

(2) $$u_{h}=\left|{\left(y\right)}_{upscale}-{\left(y\right)}_{downscale}\right|$$

(3) $$\%u_{h}{}_{max}=\frac{u_{h}{}_{max}}{r_{O}}\cdot 100=\frac{u_{hmax}}{y_{max}-y_{min}}\cdot 100$$

where $u_{h}$ is the hysteresis error, $u_{hmax}$ is the maximum hysteresis error, and $r_{O}$ is the full-scale output range, i.e., $y_{max}-y_{min}$. The hysteresis error indicates the differences between an upscale sequential test result (${\left(y\right)}_{upscale}$) and a downscale sequential test result (${\left(y\right)}_{downscale}$). In the test, ${\left(y\right)}_{upscale}$ denotes the output value when force was applied from 0 to 1 N, and ${\left(y\right)}_{downscale}$ indicates the output value when force was released from 1 to 0 N. The maximum hysteresis error was 12.57%. This hysteresis phenomenon is attributed to the elastic after-effect, where the original state is not momentarily restored even after the force is gradually released.

**Figure 12:**
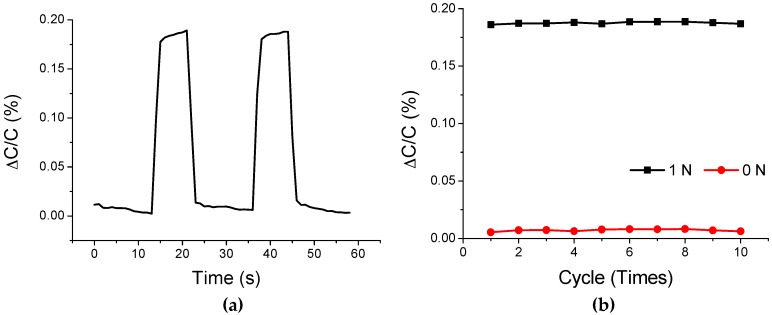
Capacitance change of the air-gap touch sensor during the repeated application of force: (**a**) switching of the input force; and (**b**) result of 10 time repetition of the switching process.

The third set of experiments was carried out to evaluate the responses of the air-gap sensor samples to the repeated application of force. A force of 1 N was applied and released at intervals of 20 s. The force was applied, maintained for 7 s, and then released. The release period was 13 s, as shown in Figure 12a. The capacitances of the sensor samples were measured while this switching process was repeated ten times. The experimental results are shown in Figure 12b.

At the first cycle, the average Δ*C*/*C* was 0.1861% when the force of 1 N was applied, and 0.0054% when the force was released. At the tenth cycle, the average Δ*C*/*C* was 0.1869% when the same force was applied and was 0.0062% when the force was released. These results show that the difference in Δ*C*/*C* between the first and tenth cycles is negligibly small, which implies that the capacitance characteristic of the sensor samples can be maintained after the repeated application of force.

## 4. Conclusions

In this study, a roll-to-roll printed electronics method was developed for the fabrication of capacitive air-gap touch sensors on a flexible substrate. Each layer of the sensor was fabricated by printing or coating. The air-gaps of the touch sensor samples were successfully formed by removing the PVA sacrificial layer. The average width and thickness of the air-gap were 10.0 mm and 113.2 μm, respectively. The measurement of electrical characteristics showed that the air-gap touch sensor samples had significantly higher sensitivities than those of the PDMS-based samples and that the hysteresis error and capacitance change during the repeated application of force were small.

Our future study will include the optimization of materials and processes to fabricate all of the layers of air-gap touch sensors with roll-to-roll printed electronics methods. Through the optimization, we expect to overcome the problem of air-gap increase during the removal process of the sacrificial layer. In addition, we will apply the proposed method to other types of sensors, such as accelerometers and gyroscopes, which are difficult to fabricate with existing printed electronics methods.

## Figures and Tables

**Table 1:** Materials and fabrication processes for the air-gap touch sensors.

Layer	Material	Process	Curing Time	Curing Temperature
Bottom electrode	Ag	Roll-to-roll slot-die coating	10 min	100 °C
Dielectric	PMMA	Roll-to-roll slot-die coating	10 min	70 °C
Sacrificial	PVA	Roll-to-roll slot-die coating	10 min	70 °C
Top electrode	Stretchable Ag	Roll-to-roll gravure printing	30 s	150 °C
Structural	Epoxy resin	Spin-coating	10 min	150 °C

## References

[1] Chang J., Zhang X., Ge T., Zhou J. (2014). Fully printed electronics on flexible substrates: High gain amplifiers and DAC. Org. Electron..

[2] Pierre A., Sadeghi M., Payne M.M., Facchetti A., Anthony J.E., Arias A.C. (2014). All-printed flexible organic transistors enabled by surface tension-guided blade coating. Adv. Mater..

[3] Willmann J., Stocker D., Dörsam E. (2014). Characteristics and evaluation criteria of substrate-based manufacturing. Is roll-to-roll the best solution for printed electronics?. Org. Electron..

[4] Perelaer J., Smith P.J., Mager D., Soltman D., Volkman S.K., Subramanian V., Korvink J.G., Schubert U.S. (2010). Printed electronics: The challenges involved in printing devices, interconnects, and contacts based on inorganic materials. J. Mater. Chem..

[5] Sekine C., Tsubata Y., Yamada T., Kitano M., Doi S. (2014). Recent progress of high performance polymer OLED and OPV materials for organic printed electronics. Sci. Technol. Adv. Mater..

[6] Lee S.H., Lee D.G., Jung H., Lee S. (2018). Application of calendering for improving the electrical characteristics of a printed top-gate, bottom-contact organic thin film transistors. Jpn. J. Appl. Phys..

[7] Lee S.H., Lee S. (2017). Using an Optimized Calendering Process with a Grey-Based Taguchi Method to Enhance the Performance of a Printed OTFT. Sci. Adv. Mater..

[8] Lee S.H., Lee S. (2018). Enhancement of the electrical performance of a printed organic thin film transistor through optimization of calendering process. Org. Electron..

[9] Lee S.H., Nguyen H.A.D., Kim J.-M., Ko S.-L., Lee S. (2016). Improvement of the Performance of Printed Organic Thin Film Transistor by Calendering Process. Sci. Adv. Mater..

[10] Montanino M., Sico G., Prontera C.T., De Girolamo Del Mauro A., Aprano S., Maglione M.G., Minarini C. (2017). Gravure printed PEDOT:PSS as anode for flexible ITO-free organic light emitting diodes. Express Polym. Lett..

[11] Wei C., Zhuang J., Zhang D., Guo W., Yang D., Xie Z., Tang J., Su W., Zeng H., Cui Z. (2017). Pyridine-Based Electron-Transport Materials with High Solubility, Excellent Film-Forming Ability, and Wettability for Inkjet-Printed OLEDs. ACS Appl. Mater. Interfaces.

[12] Li L., Pan L., Ma Z., Yan K., Cheng W., Shi Y., Yu G. (2018). All Inkjet-Printed Amperometric Multiplexed Biosensors Based on Nanostructured Conductive Hydrogel Electrodes. Nano Lett..

[13] Ali M.M., Maddipatla D., Narakathu B.B., Chlaihawi A.A., Emamian S., Janabi F., Bazuin B.J., Atashbar M.Z. (2018). Printed strain sensor based on silver nanowire/silver flake composite on flexible and stretchable TPU substrate. Sens. Actuators A Phys..

[14] Ugsornrat K., Maturos T., Pasakon P., Karuwan C., Pogfay T., Sriprachuabwong C., Wisitsoraat A., Tuantranont A. (2018). Electrowetting-on-dielectric chip with integrated screen-printed electrochemical sensor for rapid chemical analysis. Mater. Sci. Eng. B.

[15] Ko W.H., Wang Q. (1999). Touch mode capacitive pressure sensors. Sens. Actuators A Phys..

[16] Berger C., Phillips R., Pasternak I., Sobieski J., Strupinski W., Vijayaraghavan A. (2018). Touch-mode capacitive pressure sensor with graphene-polymer heterostructure membrane. 2D Mater..

[17] Roy A.L., Bhattacharyya T.K. (2015). Design, fabrication and characterization of high performance SOI MEMS piezoresistive accelerometers. Microsyst. Technol..

[18] Saqib M., Saleem M.M., Mazhar N., Awan S.U., Khan U.S. (2018). Design and Analysis of a High-Gain and Robust Multi-DOF Electro-thermally Actuated MEMS Gyroscope. Micromachines.

[19] Sonmezoglu S., Taheri-Tehrani P., Valzasina C., Falorni L.G., Zerbini S., Nitzan S., Horsley D.A. (2015). Single-Structure Micromachined Three-Axis Gyroscope with Reduced Drive-Force Coupling. IEEE Electr. Device Lett..

[20] Cheng J., Liu W., Chen Q., Xu N., Sun Q., Liu Y., Wang W., Xie H. (2018). A mems variable optical attenuator based on a vertical comb drive with self-elevated stators. Sens. Actuators A Phys..

[21] Liu H., Zhang Z., Chen J. (2018). Four-Probe Bridges for In-Line Determination of Thickness and Sidewall Etch Angle of Surface Micromachined Thin Films. Appl. Sci..

[22] Vyas A., Staaf H., Rusu C., Ebefors T., Liljeholm J., Smith A.D., Lundgren P., Enoksson P. (2018). A Micromachined Coupled-Cantilever for Piezoelectric Energy Harvesters. Micromachines.

[23] Karim M.A.U., Chung S., Alon E., Subramanian V. (2016). Fully Inkjet-Printed Stress-Tolerant Microelectromechanical Reed Relays for Large-Area Electronics. Adv. Electron. Mater..

[24] Clément P., Perez E.d., Gonzalez O., Calavia R., Lucat C., Llobet E., Debéda H. (2016). Gas discrimination using screen-printed piezoelectric cantilevers coated with carbon nanotubes. Sens. Actuators B Chem..

[25] Kim H., Kim G., Kim T., Lee S., Kang D., Hwang M.S., Chae Y., Kang S., Lee H., Park H.G., Shim W. (2018). Transparent, Flexible, Conformal Capacitive Pressure Sensors with Nanoparticles. Small.

[26] Kanazawa S., Kusaka Y., Horii Y., Ushijima H. (2018). Fully additive manufacture of a polymer cantilever with an embedded functional layer. Jpn. J. Appl. Phys..

[27] Kim J., Oh D., Kim Y., Kim T., Lee B. (2018). Printing Pressure Uniformization through Adaptive Feedforward Control in Roll-to-Roll Printing Process. http://proceedings.asmedigitalcollection.asme.org/proceeding.aspx?articleid=2715745.

[28] Kim Y., Kim M., Kim T., Kim J., Oh D. (2018). Printing pressure uniformization of a roll-to-roll system using roll runout. Microsyst. Technol..

[29] Mitra K.Y., Kapadia S., Hartwig M., Sowade E., Xu Z., Baumann R.R., Zichner R. (2018). Process Development of Large Area R2R Printing and Sintering of Conductive Patterns by Inkjet and Infra-Red Technologies Tailored for Printed Electronics. NIP Digit. Fabr. Conf..

[30] Park J., Nguyen H.A.D., Park S., Lee J., Kim B., Lee D. (2015). Roll-to-roll gravure printed silver patterns to guarantee printability and functionality for mass production. Curr. Appl. Phys..

[31] Søndergaard R.R., Hösel M., Krebs F.C. (2013). Roll-to-Roll fabrication of large area functional organic materials. J. Polym. Sci. Part B Polym. Phys..

[32] Edy R., Zhao Y., Huang G.S., Shi J.J., Zhang J., Solovev A.A., Mei Y. (2016). TiO_2_ nanosheets synthesized by atomic layer deposition for photocatalysis. Prog. Nat. Sci. Mater. Int..

[33] Fujie T., Okamura Y., Takeoka S. (2007). Ubiquitous Transference of a Free-Standing Polysaccharide Nanosheet with the Development of a Nano-Adhesive Plaster. Adv. Mater..

[34] Lee S.H., Seo H., Lee S. (2018). Fabrication of a printed capacitive air-gap touch sensor. Jpn. J. Appl. Phys..

[35] Benight S.J., Wang C., Tok J.B.H., Bao Z. (2013). Stretchable and self-healing polymers and devices for electronic skin. Prog. Polym. Sci..

[36] Deng W.-J., Wang L.-F., Dong L., Huang Q.-A. (2018). LC Wireless Sensitive Pressure Sensors with Microstructured PDMS Dielectric Layers for Wound Monitoring. IEEE Sens. J..

[37] Guanhao L., Deqing M., Yancheng W., Zichen C. (2014). Modeling and Analysis of a Flexible Capacitive Tactile Sensor Array for Normal Force Measurement. IEEE Sens. J..

[38] Lei K.F., Lee K.-F., Lee M.-Y. (2012). Development of a flexible PDMS capacitive pressure sensor for plantar pressure measurement. Microelectron. Eng..

[39] Wang X.Z., Xu T.B., Dong S.R., Li S.J., Yu L.Y., Guo W., Jin H., Luo J.K., Wu Z.H., King J.M. (2017). Development of a flexible and stretchable tactile sensor array with two different structures for robotic hand application. RSC Adv..

[40] Fank S., Demirkol M. (2006). Effect of microstructure on the hysteresis performance of force transducers using AISI 4340 steel spring material. Sens. Actuators A Phys..

[41] Li M., Chen X.L., Zhang D.F., Wang W.Y., Wang W.J. (2010). Humidity sensitive properties of pure and Mg-doped CaCu_3_Ti_4_O_12_. Sens. Actuators B Chem..

